# The Research on Path Planning Method for Detecting Automotive Steering Knuckles Based on Phased Array Ultrasound Point Cloud

**DOI:** 10.3390/s25092907

**Published:** 2025-05-04

**Authors:** Yihao Mao, Jun Tu, Huizhen Wang, Yangfan Zhou, Qiao Wu, Xu Zhang, Xiaochun Song

**Affiliations:** 1 School of Mechanical Engineering, Hubei University of Technology, Wuhan 430068, China; 2 Modern Manufacturing Quality Engineering Hubei Key Laboratory, Wuhan 430068, China

**Keywords:** nondestructive testing, ultrasonic phased array, automotive steering knuckle, ultrasonic point cloud, path planning

## Abstract

To address the challenges of automatic detection caused by the variation of surface normal vectors in automotive steering knuckles, an automatic detection method based on ultrasonic phased array technology is herein proposed. First, a point cloud model of the workpiece was constructed using ultrasonic distance measurement, and Gaussian-weighted principal component analysis was used to estimate the normal vectors of the point cloud. By utilizing the normal vectors, water layer thickness during detection, and the incident angle of the sound beam, the probe pose information corresponding to the detection point was precisely calculated, ensuring the stability of the sound beam incident angle during the detection process. At the same time, in the trajectory planning process, piecewise cubic Hermite interpolation was used to optimize the detection trajectory, ensuring continuity during probe movement. Finally, an automatic detection system was set up to test a steering knuckle specimen with surface circumferential cracks. The results show that the point cloud data of the steering knuckle specimen, obtained using phased array ultrasound, had a relative measurement error controlled within 1.4%, and the error between the calculated probe angle and the theoretical angle did not exceed 0.5°. The probe trajectory derived from these data effectively improved the B-scan image quality during the automatic detection of the steering knuckle and increased the defect signal amplitude by 5.6 dB, demonstrating the effectiveness of this method in the automatic detection of automotive steering knuckles.

## 1. Introduction

The steering knuckle is one of the key components in automotive steering assembly, and its reliability directly affects the normal operation of the vehicle and the safety of the passengers [1]. Therefore, it is crucial to conduct thorough inspections of the automotive steering knuckle before it leaves the factory. In recent years, non-destructive testing (NDT) technologies such as ultrasonic testing (UT) [2], radiographic testing (RT) [3], eddy current testing (ET) [4], magnetic particle testing (MT) [5], and penetrant testing (PT) [6] have played varying roles in the inspection of complex curved-surface structures. PT is suitable for detecting surface defects and provides an intuitive visual display of the defects, making it easier to assess. However, it is a complex and time-consuming process. MT is effective for detecting surface and near-surface defects and offers advantages such as low cost, ease of operation, high sensitivity, and high detection efficiency. However, it is less effective in detecting deep-seated defects and defects between voids. ET is also applicable for surface and near-surface defect detection and has the benefit of being non-contact, but it has certain limitations when inspecting workpieces with complex shapes. RT is ideal for detecting internal defects and provides clear imaging results. It allows accurate qualitative and quantitative evaluations and has a high detection rate for volumetric defects. However, it comes with high equipment costs, and radiation exposure is harmful to humans, requiring protective measures. UT, on the other hand, is suitable for detecting both surface and internal defects. It can quickly and accurately determine the location and size of defects, offers high sensitivity, and is applicable to defect detection in various types of components.

Compared to the methods mentioned above, ultrasonic phased array testing technology offers advantages such as high precision, high efficiency, and being harmless to the human body [7,8]. Precisely controlling the emission and reception angles of ultrasonic waves, it enables multi-angle and omnidirectional defect detection [9], making it an ideal inspection method for automotive steering knuckle inspection. Ultrasonic phased array testing technology demonstrates excellent defect detection capability for complex structural components. Xu [10] et al. focused on the defects of corner-shaped components, using linear array transducers and curved array transducers for detection through wedge coupling. Kang et al. proposed a method for defect detection in wind turbine blades using a piezoelectric flexible line sensor [11]. Hu et al. developed a flexible, elastic ultrasonic phased array patch for deep-seated damage and defects in irregularly shaped structures [12]. However, most current detection methods for automotive components still rely on manually held inspection instruments, which are inefficient. Furthermore, due to the limited attention span of operators, certain areas may be overlooked during large-scale inspections, resulting in incomplete detection results.

Automated inspection technology has garnered increasing attention due to its higher repeatability and accuracy [13,14,15]. Several methods for automated flaw detection of complex components have been proposed in recent years. Bogue Robert described the development of robotic applications in ultrasonic non-destructive testing (UNDT) [16]. Mineo Carmelo et al. proposed a synchronous acquisition system for robotic trajectory and UNDT data based on the phased array ultrasonic technique [17]. Sattar T.P. et al. designed a portable NDT robotic arm, equipped with a force sensor to keep the NDT probe normal to the surface while scanning complex shapes [18]. Guan et al. designed a region-based phased array ultrasonic total focusing method (TFM) detection system for rings with large size ranges and curved surfaces, which is capable of automatic scanning with six degrees of freedom [19]. Compared to manual inspection, automated scanning technology provides higher detection efficiency and significantly enhances the quality of the inspection results [20].

However, challenges arise in the ultrasonic inspection of curved components, as the normal direction of the part varies with changes in surface contour. To maintain a constant incident angle of the acoustic beam, the probe must continuously adjust its orientation to follow the variations in the external geometry of the curved surface. The mainstream approach is to adjust the control points along the motion path to maintain a fixed incident angle of the sound beam. Depending on whether the component’s computer-aided design (CAD) model is available, this can be divided into two main categories. When the CAD model is known, three-dimensional (3D) modeling software is used to extract points from the model and obtain the normal vector information. The corresponding control point poses are then determined through kinematic inverse solving [21,22]. However, in actual inspections, many workpieces are raw castings, and the model is unavailable. In such cases, it is necessary to build a point cloud model of the workpiece using contact or non-contact methods, followed by adjustments to the point cloud orientation [23,24]. Currently, methods for acquiring and adjusting data of workpieces with unknown models include laser scanning systems, machine vision systems, and ultrasonic scanning systems. Among these, laser scanning systems provide the most precise results (<50 μm), but their implementation is costly. Machine vision technology has moderate accuracy (0.5–5 mm) and has been successfully implemented in highly controlled environments such as conveyor-belt-type operations. However, without sophisticated training algorithms, it is difficult to determine the position of a component accurately [25]. Moreover, due to the requirement of a water-coupling environment in ultrasonic inspection, common sensors based on laser or visual inspection cannot be directly integrated. Furthermore, laser or visual inspection often requires additional external sensors, and since the coordinate system of these external devices differs from that of the inspection system, positioning errors are more likely to be introduced. In contrast, data acquisition using the ultrasonic waves generated by the ultrasonic phased array probe not only provides high precision (<1 mm) but also overcomes the challenges of detection in water. Furthermore, the system complexity is reduced since no additional hardware devices, such as lasers or vision sensors, are required for data acquisition and adjustment.

Currently, surface estimation methods based on phased array ultrasound mainly include the Pulse-Echo method, the Pitch-Catch method, the Surface Adaptive Ultrasounds (SAUL) method, and the TFM imaging method [26,27,28]. Among these methods, the Pitch-Catch method and the TFM imaging method offer high imaging accuracy and are well suited for extracting complex structural contours, while the SAUL method enables adaptive alignment of the incident wavefront with complex surfaces, theoretically allowing for more accurate interface fitting. However, both the Pitch-Catch method and the TFM imaging method require the acquisition of full matrix capture (FMC) data, which results in an extremely large volume of data when performing three-dimensional contour estimation of the entire workpiece. The SAUL method, meanwhile, requires real-time updating of delay laws, significantly increasing the computational and control complexity of the system. Considering both data acquisition efficiency and implementation feasibility, this study adopted the Pulse-Echo method as the point cloud extraction approach due to its computational simplicity and higher efficiency.

Several critical challenges remain in the implementation of automated nondestructive testing for automotive steering knuckles using ultrasonic phased array technology. Primarily, the normal direction of the steering knuckle varies with changes in the surface contour of the component, making it difficult to maintain a stable incident angle of the acoustic beam, thereby compromising the reliability of the inspection. Secondly, fluctuations in the water layer thickness increase the complexity of probe orientation adjustments. In addition, since phased array ultrasonic testing must be conducted in a water-coupled environment, it places higher demands on system integration. To address these challenges, this study employed an ultrasonic phased array probe to acquire three-dimensional point cloud data of the steering knuckle component. Based on this data, the inspection path required for automated testing was derived. Finally, experimental validation was conducted on a steering knuckle specimen containing circumferential surface cracks to evaluate the effectiveness and practicality of the proposed method.

## 2. Working Principle

### 2.1. Phased Array Ultrasonic Point Cloud Acquisition

**Figure 1:**
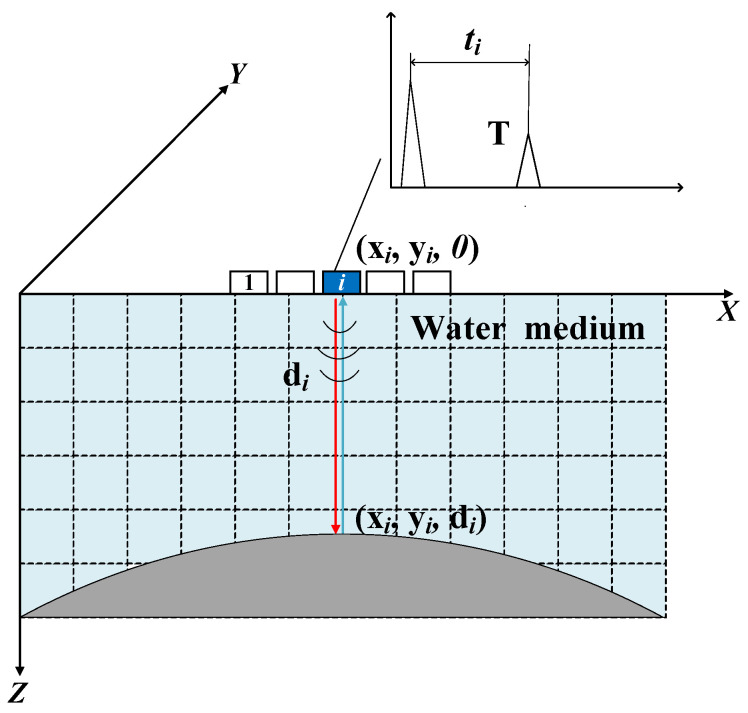
Principle of phased array ultrasonic point cloud acquisition.

**Figure 2:**
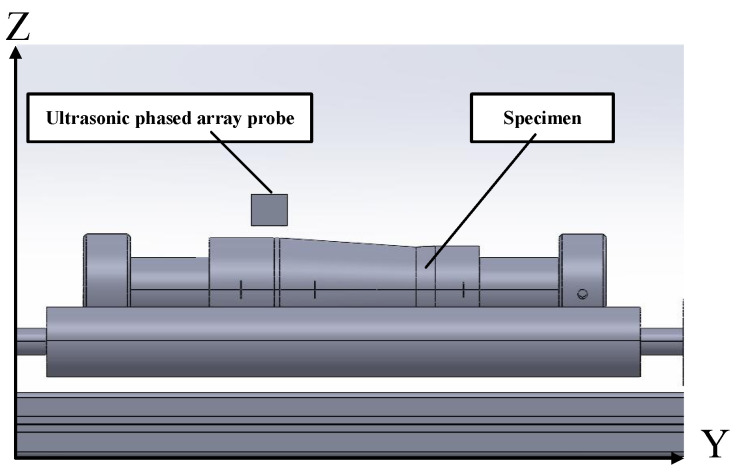
Schematic diagram of the relationship between probe and specimen position.

In this method, a water-immersion longitudinal wave technique is used to acquire the three-dimensional surface morphology of the specimen, as illustrated in Figure 1. The relative position of the probe and the specimen is shown in Figure 2. The probe and specimen are both parallel to the horizontal plane, and the distribution direction of the probe elements is perpendicular to the axis direction of the specimen. The ultrasonic phased array probe uses a self-transmitting and self-receiving mode for each element, where each array element sequentially emits ultrasonic waves, and the waves are incident on the surface of the workpiece through the water medium. At this point, part of the sound waves are reflected off the surface of the workpiece, forming a primary interface wave (T). The reflected signals from different positions vary in both time and intensity. By measuring the time difference $t_{i}$ between the transmission and reception of the ultrasonic waves from each element, the distance between the transmission point and the reflection point can be calculated using the following formula:

(1) $$d_{i}=\frac{V\cdot t_{i}}{2}$$

where $d_{i}\;$ is the distance between the transmission point and the reflection point, and V is the speed of sound in the water medium.

Based on the positions of the elements and the distances from each element to the reflection points, the spatial positions of multiple reflection points $\left(x_{i},\;y_{i},\;and\;d_{i}\right)$ can be determined, where $x_{i}$ and $y_{i}\;$ represent the specific position of the array element at the time of measurement. The automated inspection system guides the probe to acquire three-dimensional surface data of the steering knuckle. The positions of all reflection points are then consolidated into a 3D point cloud, which provides an intuitive visualization of the surface contour and structural features of the component.

### 2.2. Point Cloud Segmentation

Since the acquired 3D point cloud data typically contains a large number of points and complex spatial information, directly performing trajectory planning is very difficult and inefficient. Therefore, point cloud segmentation is required. The purpose of point cloud segmentation is to decompose the 3D point cloud data into multiple 2D slices, which simplifies the computational load and makes path planning more efficient.

**Figure 3:**
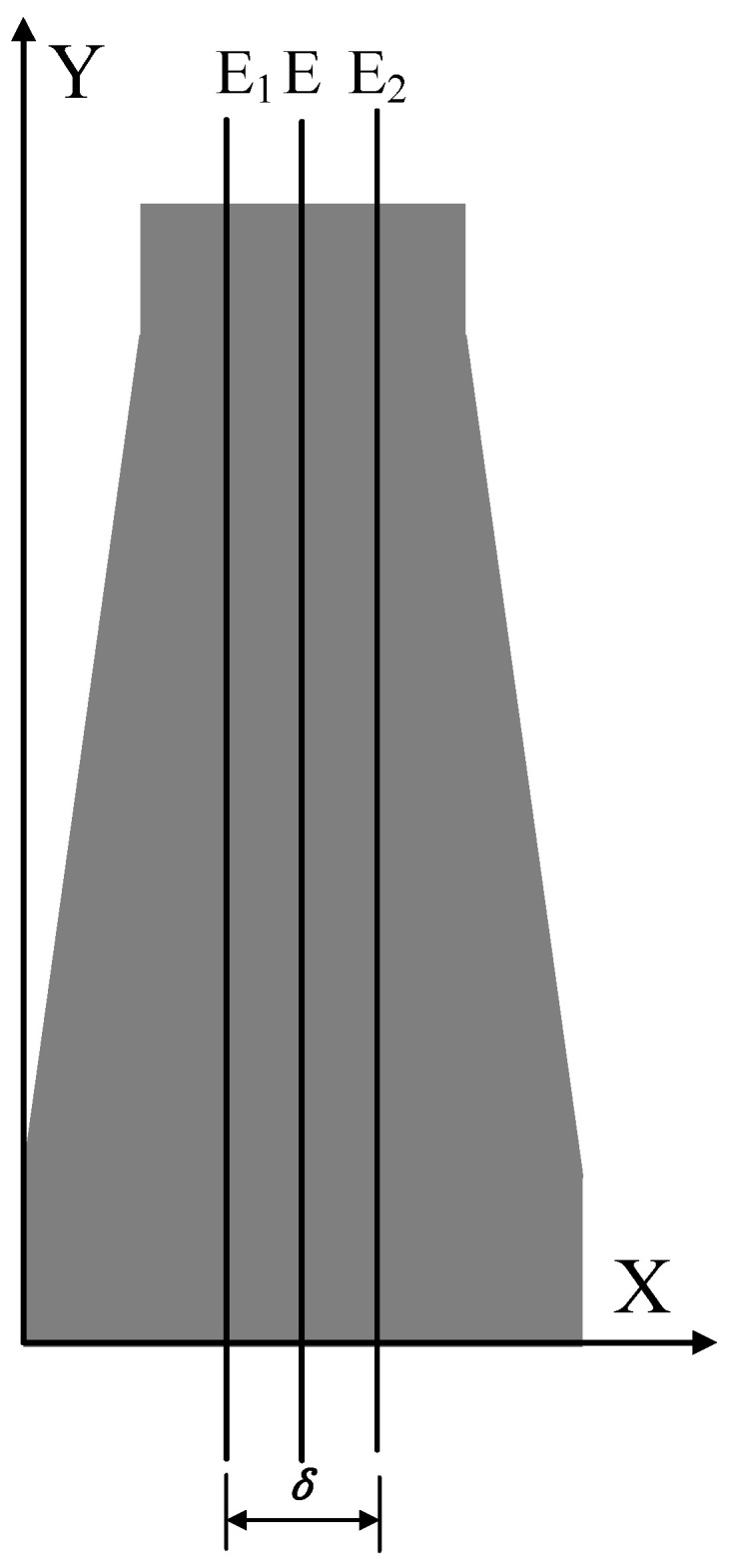
Schematic Diagram of Point Cloud Segmentation Using the Projection Method.

This paper uses the projection method for point cloud segmentation. As shown in Figure 3, the working principle of the projection method is that multiple parallel planes, spaced by δ, intersect with the workpiece’s point cloud, forming multiple slice spaces. The point cloud within each slice space is then projected onto its respective central plane E. The resulting points can serve as the desired inspection points for automated detection, providing accurate path information for subsequent path planning.

(1) Determine the slicing direction: Different slicing directions directly affect the shape of the curve in the slicing plane. This paper uses radial slicing, where the constructed planes are evenly distributed radially along the workpiece and parallel to the central axis of the point cloud;
(2) Determine the slicing thickness δ: The choice of slicing thickness δ is crucial. If δ is too large, the fitted curve will deviate from the actual surface contour of the workpiece, affecting path accuracy. If δ is too small, it may not fully represent the workpiece’s surface shape, potentially leading to overfitting of the path;
(3) Point cloud segmentation: In the point cloud segmentation process, a coordinate system is established with the slicing direction as the *x*-axis, and the slicing space is divided. By setting boundary planes $E_{1}$ and $E_{2}$, the point cloud satisfying $x_{E_{1}}\leq x\leq x_{E_{2}}$ is selected, forming a point cloud dataset $\left\{R\right\}$ that contains all point data within the slicing space. This reduces the point cloud classification problem from three dimensions to one dimension, improving classification efficiency;
(4) Distinguish the upper and lower parts: Since the point cloud in this paper is shaft-shaped, the segmented point cloud will have two parts: upper and lower. The points belonging to the upper or lower part can be distinguished by comparing the Z coordinate $Z_{i}$ of each point with the Z coordinate $Z_{mean}$ of the central axis. If $Z_{i}>Z_{mean}$, the point belongs to the upper part; if $Z_{i}<Z_{mean}$, the point belongs to the lower part.

### 2.3. Automatic Scanning Path Planning Based on Ultrasonic Point Cloud

Point cloud segmentation is performed using the projection method mentioned in Section 2.2, as shown in Figure 3. Planes parallel to the YZ plane are established at δ/2 on both sides of the central axis, and these two planes together form the slicing space, with the central plane coinciding with the central axis. The point cloud within the slicing space is projected onto the central plane, and only the upper portion of the point cloud is retained to obtain the desired inspection points. These points can be used to fit the contour curve of the top of the specimen, accurately reflecting the contour of the top of the specimen.

During the scanning process, there is a water medium layer between the phased array ultrasonic probe and the workpiece surface, which causes a distance deviation between the actual position of the probe and the inspection point due to the water layer. Additionally, without external assistance, the phased array ultrasonic probe can only move along the directions of the three XYZ axes. However, the surface being scanned is not a plane. This causes the angle between the probe and the surface to change with the variation in the surface normal during the automated detection process, resulting in a mismatch between the actual incident angle of the sound beam and the desired incident angle. This can significantly affect the quality of ultrasonic imaging. Therefore, after obtaining the coordinates of the desired inspection points, it is necessary to calculate the probe’s pose corresponding to these points. This will ensure that the water layer height between the probe and the scanned object remains constant during the scanning path and that a consistent incident angle is maintained, thereby improving the quality of the ultrasonic imaging. In this study, we used the surface normal vectors of the desired inspection points to determine the probe’s pose information.

**Figure 4:**
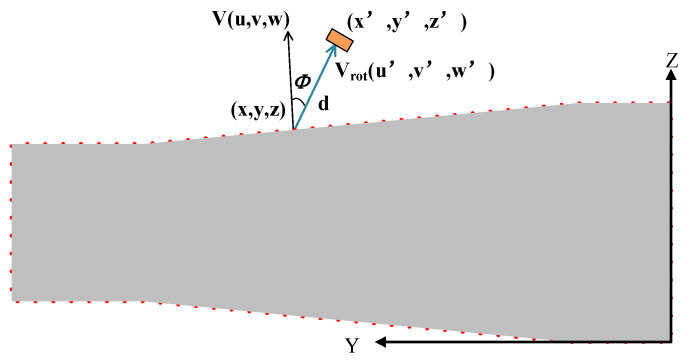
Probe pose calculation based on ultrasonic point cloud.

As illustrated in Figure 4, by rotating the normal vector of the desired inspection points by the probe’s incident angle within the plane formed by the normal vector and the *Y*-axis, the probe’s orientation direction vector can be obtained. The probe’s position is then determined by moving the inspection point along the attitude vector by the height of one water layer.

The probe’s orientation direction vector can be obtained using the Rodrigues’ rotation formula as follows [29]:

(2) $$\left\{\begin{array}{c} {\vec{\nu }}_{rot}=\vec{\nu }\cos\left(\phi \right)+\left(\vec{K}\times \vec{\nu }\right)\sin\left(\phi \right)+\vec{K}\left(\vec{K}\cdot \vec{\nu }\right)\left(1-\cos\left(\phi \right)\right)\\ \vec{K}=\vec{\nu }\times \vec{y} \end{array}\right.$$

where $\nu_{rot}$ is the rotated normal vector, which is the orientation vector of the probe; ϕ is the probe’s incident angle; K is the unit vector of the rotation axis, which is the unit normal vector of the plane formed by the desired inspection point’s normal vector and the *Y*-axis; y is the directional vector of the *Y*-axis; and ν is the normal vector of the desired inspection point.

The position and orientation vector of the probe can be calculated as follows:

(3) $$\left\{\begin{array}{c} u^{\prime }=u\cos\phi -\frac{uv}{\sqrt{w^{2}+u^{2}}}\sin\phi \\ v^{\prime }=v\cos\phi -\sqrt{w^{2}+u^{2}}\sin\phi \\ w^{\prime }=w\cos\phi -\frac{wv}{\sqrt{w^{2}+u^{2}}}\sin\phi \\ x^{\prime }=x+\frac{u^{\prime }}{\sqrt{u^{\prime 2}+v^{\prime 2}+w^{\prime 2}}}d\\ y^{\prime }=y+\frac{v^{\prime }}{\sqrt{u^{\prime 2}+v^{\prime 2}+w^{\prime 2}}}d\\ z^{\prime }=z+\frac{w^{\prime }}{\sqrt{u^{\prime 2}+v^{\prime 2}+w^{\prime 2}}}d \end{array}\right.$$

where x,  y,  and z are the coordinates of the desired inspection points; u, v, and w are the components of the normal vector along the X, Y, and Z axes, respectively. $x^{\prime },\;y^{\prime },\;\;and\;z^{\prime }$ are the probe position coordinates corresponding to the desired scanning point. $u^{\prime },v^{\prime },\;and\;w^{\prime }$ are the components of the probe attitude vector along the X, Y, and Z axes, respectively; d is the water layer height.

**Figure 5:**
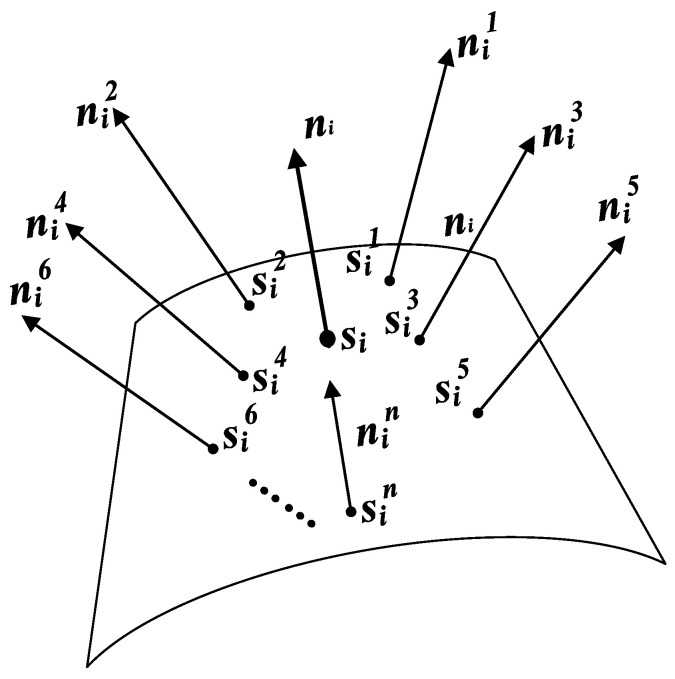
Schematic diagram of neighboring points and normal vectors on a curved surface.

Classic methods for calculating the normal vector of a point in a point cloud include the least squares fitting method, RANSAC plane fitting method, and principal component analysis (PCA), with PCA being the most widely used method [30]. The least squares fitting method is prone to errors when processing point clouds with significant noise, resulting in inaccurate calculations. Although the RANSAC plane fitting method can effectively eliminate the impact of outliers, it typically requires multiple iterations to achieve satisfactory fitting results for large-scale point cloud data, which leads to high computational complexity. Additionally, the RANSAC method may exhibit certain limitations when handling point cloud data with complex surface structures [31]. In contrast, PCA can effectively capture the dominant direction of the point cloud by performing eigenvalue decomposition on the covariance matrix of the neighboring point set within a specific region of the point cloud, thus avoiding the complex iterative optimization processes [32]. It is able to directly extract the normal vector of a point in the point cloud and maintain high computational accuracy even in the presence of noise, offering a distinct advantage in normal vector calculation [33]. Taking Figure 5 as an example, the normal vector of any point $s_{i}$ in the point cloud can be estimated using its k nearest neighbors. The k-d tree [34] is used in this research to search for the k-nearest neighbors around $s_{i}$; the k-nearest points are noted as $s_{i}^{n}(n\in \left[1,k\right])$. The covariance matrix is constructed from $s_{i}$ and its k nearest neighbors $s_{i}^{n}(n\in \left[1,k\right])$:

(4) $$E_{3\times 3}=\frac{1}{k}\sum_{n=1}^{k}\left(s_{i}^{n}-{\bar{s}}_{i}\right)\cdot {\left(s_{i}^{n}-{\bar{s}}_{i}\right)}^{T}$$

where ${\bar{s}}_{i}$ is the geometric center calculated from the *k* nearest neighbors, and $E_{3\times 3}$ is a symmetric, non-negative definite matrix. The eigenvalues and eigenvectors of $E_{3\times 3}$ are given by the following equation:

(5) $$E_{3\times 3}\cdot \vec{\nu_{l}}=\lambda_{l}\cdot \vec{\nu_{l}},l\in \left\{1,2,3\right\}$$

where $\lambda_{1}$, $\lambda_{2}$, and $\lambda_{3\;}(\lambda_{1}<\lambda_{2}<\lambda_{3})$ are the eigenvalues, and $\vec{\nu_{1}}$, $\vec{\nu_{2}}$, and $\vec{\nu_{3}}$ are the corresponding eigenvectors. The eigenvector corresponding to the smallest eigenvalue can be estimated as the normal vector of the point $s_{i}$ in the point cloud.

Due to the uneven distribution of the acquired ultrasonic point cloud, directly applying PCA to calculate the normal vectors may not yield accurate results. This is because points in dense regions have an excessive influence on the analysis, while points in sparse regions contribute insufficiently. To address this issue, a Gaussian-weighted PCA method is proposed to more accurately calculate the normal vectors. This method assigns Gaussian-distributed weights to each neighboring point in the point cloud, ensuring that closer neighbors have a greater contribution to the result and thus more accurately reflecting the local geometric features of the point cloud. The use of Gaussian weights smoothly attenuates the influence of points farther from the center, effectively reducing the interference of noise points and uneven point distribution in the normal vector calculation.

The centering step of Gaussian-weighted PCA is similar to that of standard PCA, but the weighted centroid is considered when calculating the centroid. The centering step involves calculating the weighted centroid:

(6) $$\left\{\begin{array}{c} {\bar{s}}_{i}=\frac{\sum_{n=1}^{k}\omega_{n}s_{i}^{n}}{\sum_{n=1}^{k}\omega_{n}}\\ \omega_{n}=\exp\left(-\frac{d_{n}^{2}}{2\sigma^{2}}\right) \end{array}\right.$$

where $\omega_{n}$ is the Gaussian weight, $d_{n}$ is the Euclidean distance between the current point and its neighbor, and σ is the standard deviation of the Gaussian distribution, which controls the rate of weight decay. A smaller σ causes the weight to decay quickly, thereby making the influence of points that are closer more significant, while a larger σ causes the weight to decay more slowly, allowing points farther away to have a greater influence on the result.

In Gaussian-weighted PCA, the covariance matrix must also be adjusted according to the weights. The formula for calculating the weighted covariance matrix is as follows:

(7) $$E_{3\times 3}=\frac{1}{\sum_{n=1}^{k}\omega_{n}}\sum_{n=1}^{k}\omega_{n}\left(s_{i}^{n}-{\bar{s}}_{i}\right)\cdot {\left(s_{i}^{n}-{\bar{s}}_{i}\right)}^{T}$$

Similar to standard PCA, the weighted covariance matrix is subjected to eigenvalue decomposition. The eigenvector corresponding to the smallest eigenvalue can be estimated as the normal vector of the point $s_{i}$ in the point cloud.

### 2.4. Path Optimization Based on Cubic Hermite Interpolation

**Figure 6:**
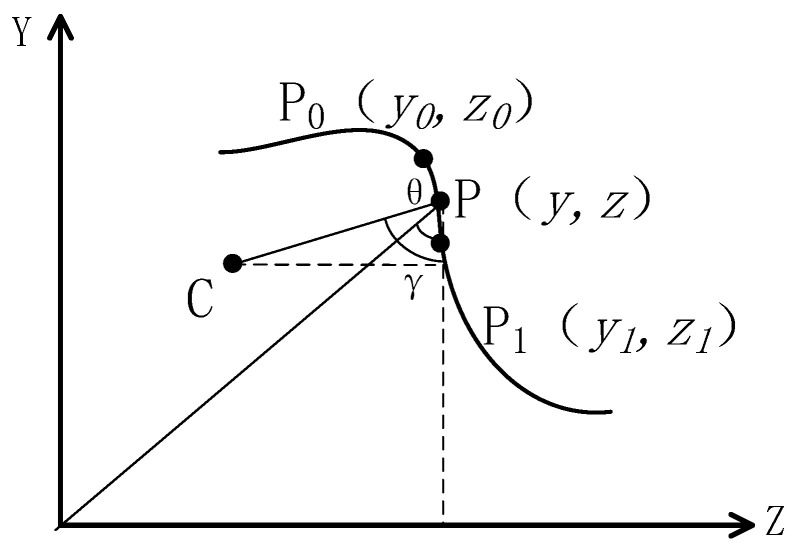
Path optimization based on cubic Hermite interpolation.

Since the surface of the object being inspected is not perfectly smooth, to avoid abrupt changes in the path and ensure the continuity and smoothness of the movement, this study used piecewise cubic Hermite interpolation to optimize the path [35], as illustrated in Figure 6.

For a curve S located in the Y-Z plane, each point of curvature on S corresponds to a center of curvature C. The curvature point P(x,z) is connected to both the center of curvature C and the origin O, forming two right-angled triangles. Let γ be the angle between the normal of the curvature point and the vertical direction and θ the arctangent of the ratio of the vertical and horizontal coordinates of the curvature point. As the curve S changes, both γ and θ change synchronously, and there is a corresponding relationship between them. A piecewise cubic Hermite interpolation function is used to fit the correspondence between these two variables, as shown by the following formula:

(8) $$V\left(\theta \right)=\gamma_{a}A\left(\theta \right)+\gamma_{b}B\left(\theta \right)+\gamma_{a}^{\prime }C\left(\theta \right)+\gamma_{b}^{\prime }D\left(\theta \right)$$

(9) $$\theta_{i}=\arctan\left(\frac{z_{i}}{y_{i}}\right)$$

where $\gamma_{a}$ and $\gamma_{b}$ are the normal vector values at points $P_{0}$ and $P_{1}$. $\gamma_{a}^{\prime }$ and $\gamma_{b}^{\prime }$ are the derivative values of the normal vectors at $P_{0}$ and $P_{1}$, representing the rate of change of the normal vector at these points. A(θ), B(θ), C(θ), and D(θ) are expressions involving the powers of θ in the interpolation function. The calculation formulas for these terms are as follows:

(10) $$\left\{\begin{array}{c} A\left(\theta \right)=1+2\frac{\theta -\theta_{0}}{\theta_{1}-\theta_{0}}{\left(\frac{\theta -\theta_{1}}{\theta_{0}-\theta_{1}}\right)}^{2}\\ B\left(\theta \right)=1+2\frac{\theta -\theta_{1}}{\theta_{0}-\theta_{1}}{\left(\frac{\theta -\theta_{0}}{\theta_{1}-\theta_{0}}\right)}^{2}\\ C\left(\theta \right)=\left(\theta -\theta_{0}\right){\left(\frac{\theta -\theta_{1}}{\theta_{0}-\theta_{1}}\right)}^{2}\\ D\left(\theta \right)=\left(\theta -\theta_{1}\right){\left(\frac{\theta -\theta_{0}}{\theta_{1}-\theta_{0}}\right)}^{2} \end{array}\right.$$

The coordinates of the interpolation points are expressed as follows:

(11) $$\left\{\begin{array}{c} y\left(\theta \right)=y_{0}\left(\frac{\theta_{1}-\theta }{\theta_{1}-\theta_{0}}\right)+y_{1}\left(\frac{\theta -\theta_{0}}{\theta_{1}-\theta_{0}}\right)\\ z\left(\theta \right)=z_{0}\left(\frac{\theta_{1}-\theta }{\theta_{1}-\theta_{0}}\right)+z_{1}\left(\frac{\theta -\theta_{0}}{\theta_{1}-\theta_{0}}\right) \end{array}\right.$$

After obtaining the normal vectors of the interpolation points, the optimized path points and their attitude direction vectors can be determined by applying Formula (3) from Section 2.3, along with the probe’s incident angle and water layer height. This process ensures that the position and direction of the path points in space are aligned with the variation of the normal vectors, achieving more accurate path planning.

### 2.5. Path Planning Steps

**Figure 7:**
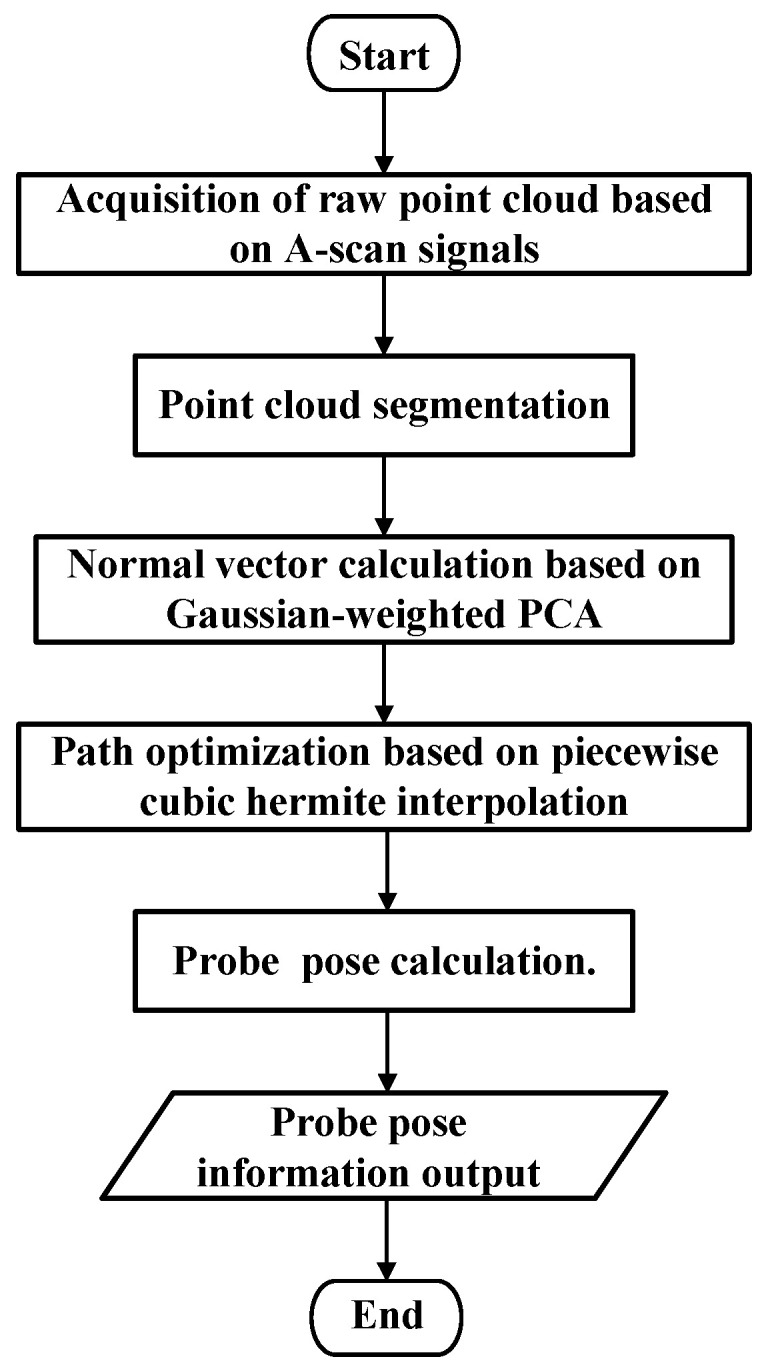
Steps for scanning path planning based on ultrasonic point cloud.

Figure 7 outlines all the steps of ultrasound-assisted path planning, which include the following:

Step 1: The collected A-scan signals are input, the first interface wave of each acquisition point’s array element signal is extracted, and the vertical distance from each array element to the workpiece is calculated to obtain the raw point cloud data.

Step 2: Point cloud segmentation is performed using the projection method described in Section 2.2. By establishing slicing planes parallel to the YZ plane on both sides of the central axis, the slicing space is determined. All points within the space are projected onto the central plane, and the upper portion of the points is selected as the desired inspection points.

Step 3: The normal vector for each desired inspection point is calculated using Gaussian-weighted principal component analysis (PCA).

Step 4: A piecewise cubic Hermite spline interpolation method is employed to smooth the abrupt sections of the trajectory points, resulting in interpolated point cloud data and their corresponding normal vectors.

Step 5: The normal vectors of each point cloud are rotated, and the points are moved along the rotated vectors by a water layer distance to obtain the probe’s pose information.

## 3. Experimental Configuration

**Figure 8:**
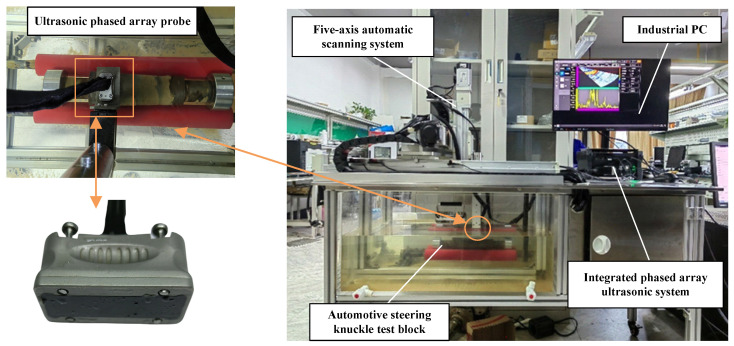
Water-immersion phased array ultrasonic testing system.

**Figure 9:**
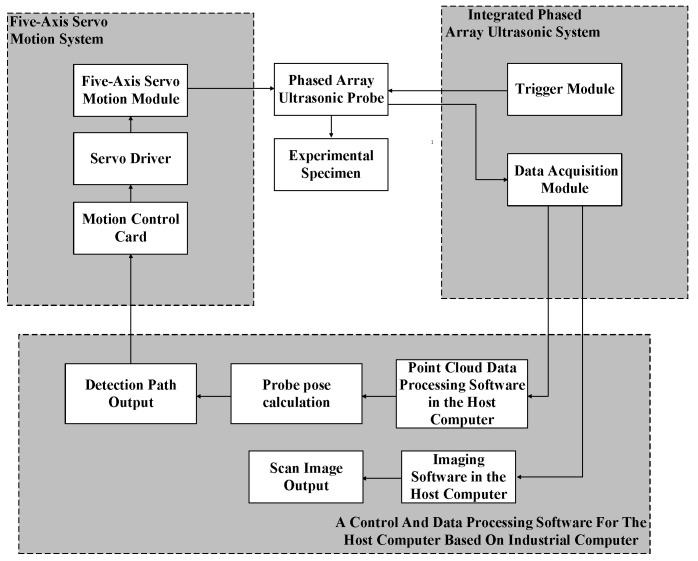
System schematic diagram.

To verify the effectiveness of the path planning algorithm proposed in this paper, a water-immersion phased array ultrasonic testing system was developed, as shown in Figure 8. The system consists of four main components: a five-axis servo motion system, an Eintik PHASELINK integrated phased array ultrasonic system, an Eintik M12-10L64 phased array ultrasonic probe, and a control and data processing software for the host computer based on industrial computer. The automatic scanning system is composed of a three-degree-of-freedom scanning mechanism, a workpiece rotary motion mechanism, and a probe rotating motion mechanism. The principle of the testing system is shown in Figure 9, and the main parameters are listed in Table 1.

The workflow of the system is as follows:

(1) Point cloud data acquisition: The dimensions of the steering knuckle and motion parameters are input. The motion control card transmits parameters such as scanning mode, speed, and range to the servo motor driver. The *Y*-axis servo motor drives the probe to perform axial step scanning on the steering knuckle, while the workpiece rotary motor drives the workpiece to perform step rotational movement. Both axes work in coordination to complete the scanning task of the workpiece;
(2) Point cloud data collection of the steering knuckle: During the collection of point cloud data, the multi-axis motion control card transmits the current actual position of the probe in real-time to the upper computer’s data acquisition software. When the predetermined position is reached, ultrasonic A-scan data are collected. The pre-processed inspection data and position information are processed by the point cloud collection software to generate the point cloud image and save the point cloud data;
(3) Automatic path generation and inspection: The probe pose information of each point on the scanning path is input into the upper computer software of the automatic scanning system to complete the automated path scanning. During the execution, the probe collects ultrasonic A-scan signals and transmits the A-scan signals along with the position information in real time to the upper computer’s imaging software. After processing, B-scan images are generated.

## 4. Experimental Verification and Analysis

**Figure 10:**
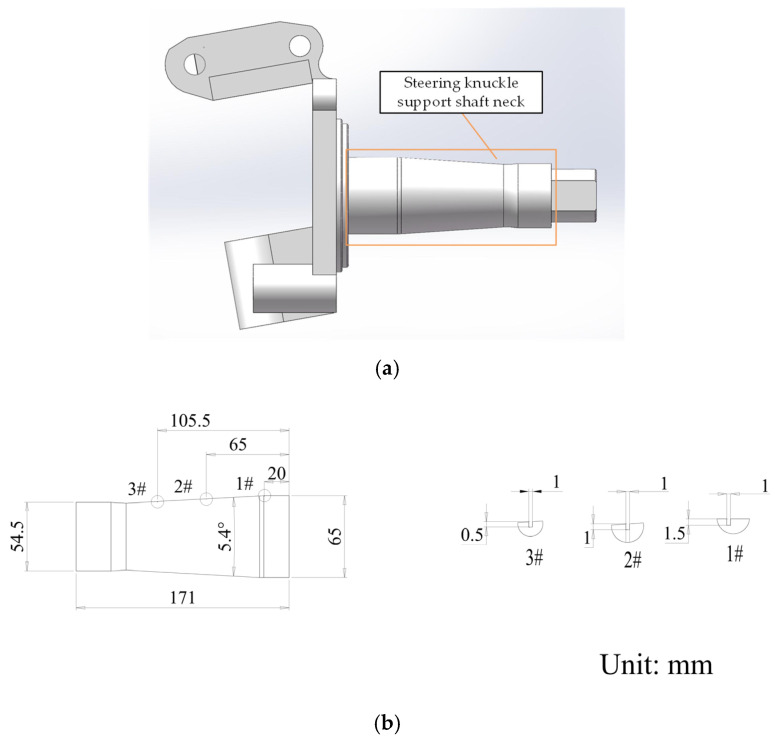
Experimental specimen. (**a**) Schematic diagram of the support shaft neck of the automotive steering knuckle. (**b**) Partial drawing of the specimen.

**Figure 11:**
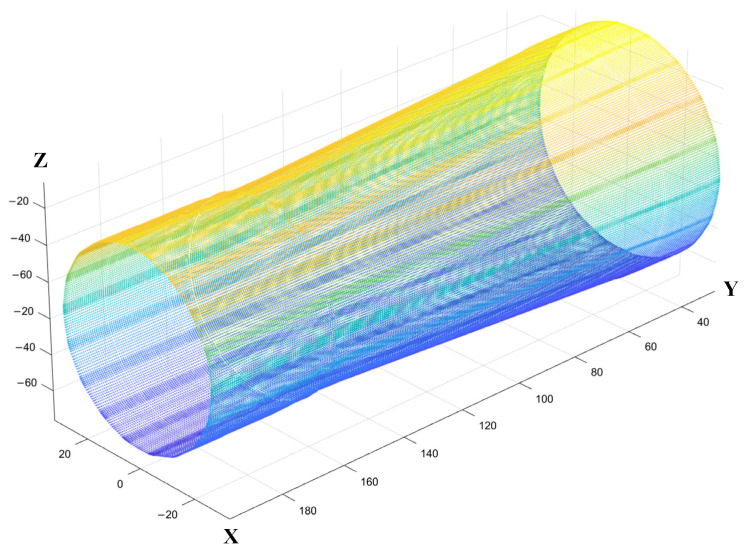
Automotive steering knuckle specimen point cloud image.

In this study, a tapered shaft specimen, as shown in Figure 10, was used to replace the steering knuckle support shaft neck for testing. The specimen was made with a one-to-one scale based on the 3D dimensions of the automotive steering knuckle support shaft neck and machined with three grooves. The probe is mounted on the clamping device of the *Z*-axis, as shown in Figure 8, and the automatic scanning system drives the probe to scan the specimen and collect relevant data. During the experiment, the system first drives the probe to perform scanning. Due to the limited detection range of the probe, each scan only covers a portion of the specimen. Then, the workpiece rotation mechanism drives the specimen to rotate around its axis to the set angle, allowing the undetected area to enter the probe’s scanning range. The system then drives the probe to perform another scan and repeats the process until the entire specimen is scanned. The probe moves 0.6 mm for each sampling, with a sampling interval of 0.1 s, ensuring consistent time and spacing between each sampling. The step angle of the specimen’s rotation axis is set to 20°, meaning that after each scan, the specimen will rotate 20° to enter the next scan. Due to the large incident angles of the sound waves excited by the probe’s elements at both ends relative to the specimen’s surface, this can affect the accuracy of point cloud measurements. Therefore, only the 24 central elements of the probe were used for detection in each scan. These elements correspond to the region at the top of the specimen where the reflection angle is smaller. With a smaller reflection angle in the detection area, most of the ultrasonic energy returns along the original path, and only a small amount of energy is reflected in other directions. This selection helps avoid interference from the excessively curved edge areas, ensuring higher measurement accuracy. Additionally, using the first-interface echo to collect point cloud data helps obtain direct surface reflection signals, reducing the impact of curvature on subsequent reflection signals and more accurately capturing the surface morphology of the specimen. The effective detection length of the middle 24 elements of the probe is 14 mm. The collected ultrasonic data were processed to generate the point cloud image shown in Figure 11. In the point cloud processing and subsequent scan path point calculations, all computations were carried out in a global coordinate system with the center of the probe’s initial scan position as the origin. This approach simplifies the coordinate transformation process and reduces computational complexity.

**Figure 12:**
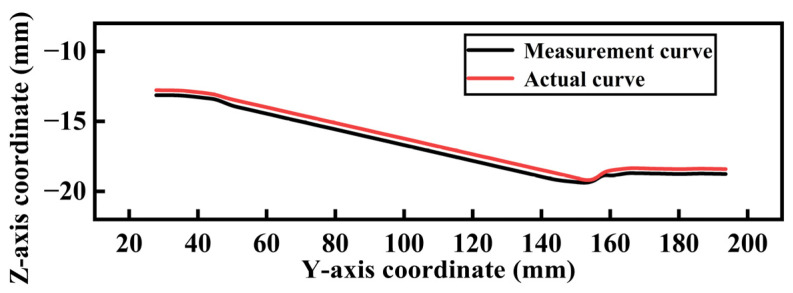
Comparison chart of measured and actual curves.

Point cloud segmentation was performed using the projection method described in Section 2.2. Planes parallel to the YZ plane were established at δ/2 on both sides of the central axis, and these two planes together define the slicing space, with the central plane coinciding with the central axis. The value of δ is based on the average nearest neighbor distance of the point cloud $\delta_{0}$. To estimate this average distance, 100 points were randomly selected from the point cloud dataset. For each point, the distances to its 10 nearest neighbors were calculated. The average of these neighbor distances across all selected points was computed to obtain the average nearest neighbor distance for the entire point cloud. When the average nearest neighbor distance $\delta_{0}$ of the point cloud was 1.26 mm, δ was set to 1.3 mm. The point cloud within the slicing space was projected onto the central plane, thereby obtaining the surface contour points of the top of the point cloud. By connecting these points, the top contour curve of the automotive steering knuckle point cloud image was obtained, which was then compared with the actual top contour curve of the specimen, as shown in Figure 12. The three defect locations were used as measurement points, and simultaneously, with the right end surface as the reference, one measurement point was selected every 10 mm. By comparing the axial diameters of the point clouds at different locations with the actual shaft neck, the corresponding results were obtained, as detailed in Table 2. The maximum absolute error between the measured axial diameter and the actual axial diameter of the specimen did not exceed 0.8 mm, and the relative error did not exceed 1.4%.

**Figure 13:**
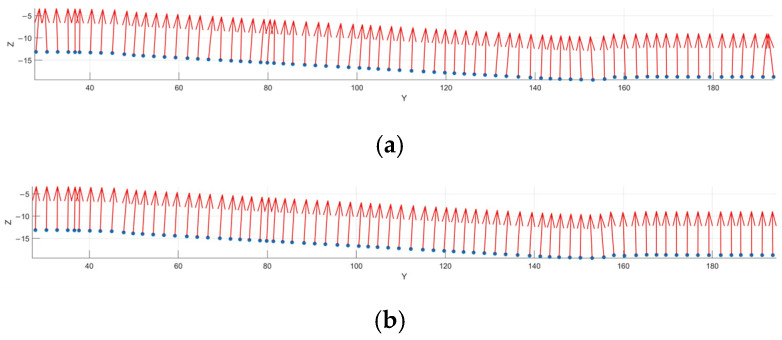
Comparison of Two PCA Normal Vector Calculation Methods. (**a**) Normal Vector Calculation Result Using Standard PCA Method. (**b**) Normal Vector Calculation Result Using Gaussian-Weighted PCA Method.

**Figure 14:**
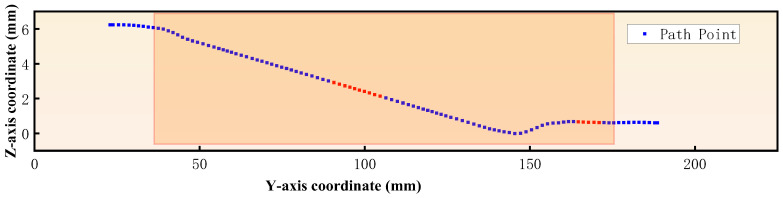
Scanning path. *The red points correspond to the positions for which the probe poses are listed in Table 3*.

The top contour points mentioned above were used as the desired inspection points. By employing the k-d tree search algorithm, the 50 nearest neighbors for each desired inspection point were identified. Subsequently, the normal vectors were calculated using both the standard PCA and Gaussian-weighted PCA methods, where the standard deviation *σ* of the Gaussian weight function was 0.5, as shown in Figure 13. As shown in the figure, some of the normal vectors calculated using standard PCA were not perfectly perpendicular to the point cloud’s contour, whereas the normal vectors calculated using Gaussian-weighted PCA were perpendicular to the point cloud’s contour. This result indicates that using Gaussian-weighted PCA for normal vector calculation provides a more accurate normal vector estimation compared to the standard PCA method. Then, a piecewise cubic Hermite interpolation method was used to interpolate the abrupt sections of the desired path and their corresponding normal vectors, resulting in interpolated points and their associated normal vectors. Finally, based on Equation (3), the detection path shown in Figure 14 was calculated by the original points, interpolated points, and their normal vectors.

The actual probe angles were calculated for the points on the path based on their attitude vectors, with some of the calculated results for the points marked in red in Table 3. The incident angle between the ultrasonic phased array probe and the specimen surface was set to always be 17°, with a water layer height of 37 mm. Since the taper angle of the specimen at the reduced shaft diameter section was 2.7°, the theoretical angle of the probe when detecting the diameter transition section was 19.7°. As shown in the table, the actual calculated values differed from the set values by no more than 0.5°, which had no impact on the detection results. In Table 3, The “calculated angle” refers to the probe angles at each path point, which was calculated using the method presented in this paper. The “theoretical angle” refers to the probe angle directly calculated based on the geometric data of the test block to ensure the incident angle of the sound beam.

**Figure 15:**
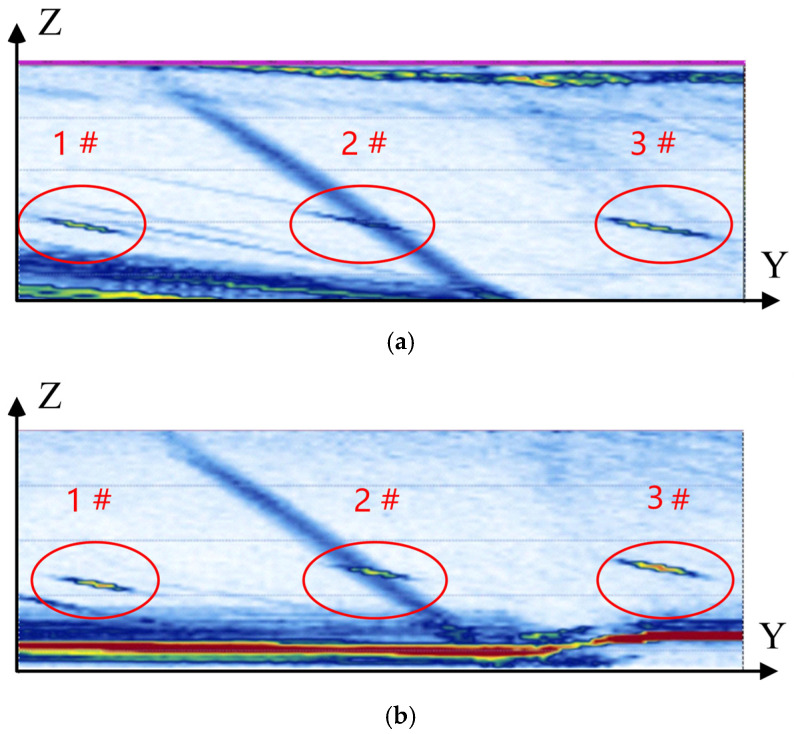
Automotive steering knuckle specimen B-scan inspection image. (**a**) Linear scanning. (**b**) Path scanning.

To compare the advantages of the method proposed in this paper, the B-scan images of the boxed area in Figure 14 were obtained using two scanning methods: a fixed 17° angle linear scan and a scan along the planned trajectory. During the scan, a linear scanning type was used with an aperture element number set to 16. The water layer was set as a 20-degree wedge, and the sound velocity in the wedge was set to the longitudinal wave velocity of water. The focusing laws for different media, provided by the instrument, were applied to compensate for the refraction occurring on the surface of the shaft. Meanwhile, the focus depth of the sound beam was set to 80 mm. The images are shown in Figure 15. The experimental results show that, compared to straight-line scanning, the method proposed in this paper not only makes the artificial defects in the B-scan images clearer but also effectively improves the spatial distribution of the defects in the images. Specifically, the image obtained from scanning along the planned path shows an inclined distribution of defects, with defects positioned lower on the left and higher on the right, which aligns with the actual spatial distribution of defects in the specimen. In contrast, the image obtained from the linear scan shows defects with a uniform depth distribution, failing to accurately reflect the actual spatial location and distribution of the defects. The fundamental reason for this difference lies in the variation of the water layer thickness during the linear scan. Since the specimen has a variable diameter structure, as the probe moves along the scan path, the water layer thickness gradually increases. The slower propagation speed of sound waves in water leads to a longer propagation time as the water layer thickens. This results in the calculated defect depth being overestimated, causing a distortion of the real spatial distribution of defects and failing to accurately reflect the actual location of defects. On the other hand, the method of scanning along the planned path ensures a stable water layer height, effectively avoiding the impact of water layer variation on sound wave propagation, thus presenting a more accurate representation of the true distribution of defects.

**Figure 16:**
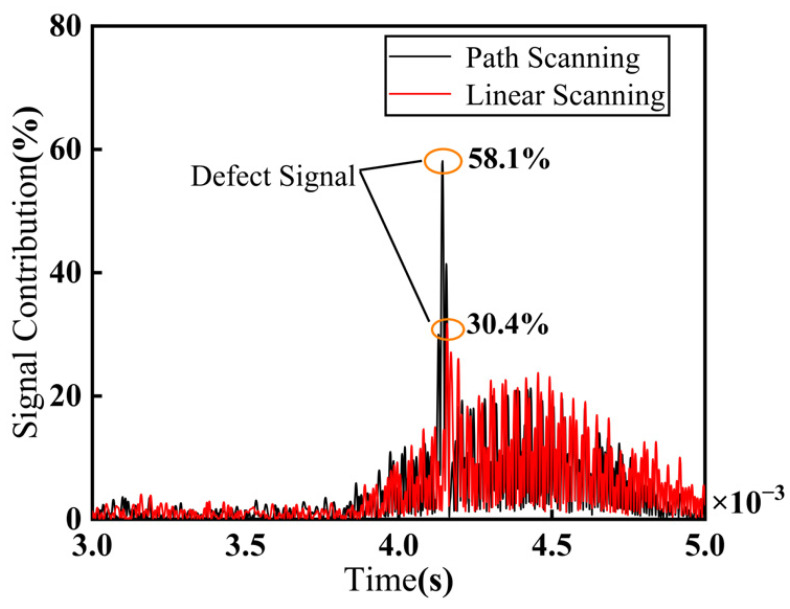
Comparison of A-scan signals for defect positions.

The A-scan signals at the defect location 2# from the B-scan images obtained using the two methods were extracted, as shown in Figure 16. When a 45 dB gain was applied, the defect signal obtained from the trajectory scan occupied 58.1% of the full-screen signal, while the defect signal obtained from the linear scan occupied 30.4% of the full-screen signal. The results in the figure show that the method described in this paper significantly enhances the amplitude of the defect signal, with an increase of 5.6 dB. This phenomenon is because the geometry of the part with varying shaft diameter causes a deviation between the actual incident angle and the probe angle. This results in the ultrasonic wave propagation path not always being optimal during the linear scan, leading to weaker echo signal strength. However, the trajectory scan method adjusts the probe orientation to maintain a constant incident angle, allowing the ultrasonic signal to propagate at a more suitable angle, thereby improving the defect echo signal intensity.

## 5. Discussion

The point cloud measurement method used in this study is based on the Pulse-Echo mode, offering the advantages of simple computation and high measurement efficiency. However, it has certain limitations when applied to parts with large curvature. Although not explicitly presented in the experimental section, it was observed during the experiments that when the incident angle of the sound beam relative to the specimen surface was controlled within 15°, the measurement results of the distance between the transmission point and the reflection point were highly accurate. When the incident angle was between 15° and 30°, the measurement error gradually increased with the incident angle. When the incident angle exceeded 30°, significant scattering of the sound beam occurred on the specimen surface, leading to considerable distortion in the measurement results.

The sources of error in this method include the limitations of the longitudinal resolution of ultrasound. The 10 MHz probe used in this study has a longitudinal resolution of 0.15 mm, which means that ultrasound waves cannot distinguish subtle variations smaller than this resolution, potentially leading to reduced measurement accuracy. In addition, variations in the speed of sound in water also had an impact on distance calculations. Factors such as water temperature, pressure, and impurity content can cause fluctuations in the speed of sound. If the speed of sound in water is not calibrated in real-time during measurements, it may lead to errors in distance measurements. Furthermore, systematic errors are also one of the main sources of point cloud measurement errors. In actual measurements, if the probe and the workpiece are not perfectly level, it may cause a deviation in the incident angle between the probe and the workpiece surface, which in turn affects the propagation path of the ultrasonic waves and measurement accuracy. An inaccurate relative position between the probe and the workpiece could introduce additional geometric errors, thus affecting the measurement accuracy. To reduce these errors, the system must be calibrated accurately. Due to the influence of the ultrasonic incidence angle on point cloud measurement, for shaft workpieces, the workpiece can be rotated to ensure that the reflection angles within the detection area are small, thereby enabling accurate measurement of the workpiece surface point cloud. For non-shaft workpieces, this method is more suitable for workpieces with small curvature and relatively flat surfaces. However, for workpieces with larger curvature, the propagation and reflection of ultrasonic waves may be significantly disturbed, which could affect the measurement accuracy. Therefore, the applicability of this method may be limited in such cases.

## 6. Conclusions

This research involved an in-depth study on two issues during the automatic scanning process of automotive steering knuckles: the inability to maintain a constant incident angle of the sound beam during detection and the variation in water layer thickness. Firstly, a point cloud acquisition method based on phased array ultrasonic technology is herein proposed. By using phased array ultrasound to measure the point cloud, three-dimensional data acquisition of the workpiece surface and defect detection were performed with the phased array probe, solving the issue that traditional 3D point cloud data acquisition methods cannot be applied in water-coupled environments. This method does not require additional hardware devices, such as lasers or vision sensors, thereby reducing the complexity of the system and detection costs. Secondly, by combining the water layer height, probe incident angle, and ultrasonic point cloud normal vectors, a correlation model between probe posture information and the point cloud was established. Based on this, a Gaussian-weighted PCA method was employed to estimate the normal vectors of the point cloud, thereby eliminating the computational errors caused by uneven point cloud distribution in traditional PCA when estimating normal vectors and improving the accuracy of normal vector estimation. Thirdly, a path optimization method based on piecewise cubic Hermite interpolation was used to refine the scanning path, ensuring smooth and continuous probe movement throughout the process. Finally, to verify the effectiveness of the proposed method, an automotive steering knuckle phased array ultrasonic automatic detection system was built, and a specimen of the automotive steering knuckle was prepared. Through full circumferential and full-length scanning of the specimen, point cloud data of the specimen were acquired, and the point cloud’s axial diameter was compared with the actual axial diameter, with the relative measurement error controlled within 1.4%. Based on these data, an automatic detection path for the phased array probe’s flaw detection was calculated. Subsequently, B-scan automatic detection was performed on the steering knuckle specimen. The results show that the proposed method can effectively optimize the B-scan quality for automotive steering knuckle automatic detection while increasing the defect signal amplitude by 5.6 dB, thus fully validating the effectiveness of the method in automotive steering knuckle automatic detection.

## Figures and Tables

**Table 1:** System Components and Parameters.

Components	Vendor	Parameters
Integrated Phased Array Ultrasonic System	Eintik	Sampling frequency: 100 MHz
Phased Array Ultrasonic Probe	Eintik	64 elements, center frequency 10 MHz; center-to-center distance 0.6 mm; element width 0.5 mm
Three-Axis Gantry Sliding Table	Leading Technology	XYZ Travel: 600, 1000, 400 mm; max speed: 500 mm/s; positioning accuracy: 0.01 mm
Probe Rotation Mechanism	Self-made	Minimum controllable angle: 0.1°; rotation axes: X
Workpieces Rotation Mechanism	Self-made	Minimum controllable angle: 0.1°; rotation axes: Y

**Table 2:** Point cloud error analysis table.

Point	Point Cloud Axial Diameter (mm)	Actual Axial Diameter (mm)	Absolute Error (mm)	Relative Error
1#	64.65	65	0.35	0.54%
2#	58.76	59.41	0.65	1.09%
3#	54.42	55.16	0.74	1.34%
1	64.70	65	0.30	0.47%
2	64.64	65	0.36	0.56%
3	64.05	64.31	0.26	0.41%
4	62.52	62.73	0.21	0.34%
5	61.38	62.07	0.69	1.12%
6	60.25	61	0.75	1.24%
7	59.18	60	0.82	1.38%
8	58.05	58.55	0.50	0.87%
9	56.96	57.63	0.67	1.18%
10	55.93	56.22	0.28	0.50%
11	54.95	55.68	0.72	1.31%
12	53.85	54.55	0.70	1.34%
13	53.64	53.71	0.07	0.14%
14	53.47	53.71	0.24	0.45%
15	53.78	54.5	0.72	1.32%
16	54.23	54.5	0.27	0.50%
17	54.17	54.5	0.33	0.62%
18	53.83	54.5	0.67	1.24%

**Table 3:** Path Point Probe Calculated Angle vs. Theoretical Angle Comparison.

Point	Coordinates	Attitude Vector	Calculated Angle	Theoretical Angle	Error
1	(0, 90.67, 2.92)	(0, 0.3451, 0.9385)	20.19°	19.7°	0.49°
2	(0, 92.34, 2.82)	(0, 0.3452, 0.9385)	20.19°	19.7°	0.49°
3	(0, 93.96, 2.73)	(0, 0.3452, 0.9385)	20.18°	19.7°	0.48°
4	(0, 95.48, 2.65)	(0, 0.3451, 0.9385)	20.19°	19.7°	0.49°
5	(0, 96.94, 2.57)	(0, 0.3451, 0.9385)	20.19°	19.7°	0.49°
6	(0, 98.40, 24.89)	(0, 0.3449, 0.9386)	20.17°	19.7°	0.47°
7	(0, 99.85, 2.41)	(0, 0.3446, 0.9387)	20.16°	19.7°	0.46°
8	(0, 101.35, 2.32)	(0, 0.3444, 0.9389)	20.14°	19.7°	0.44°
9	(0, 102.95, 2.23)	(0, 0.3435, 0.9391)	20.09°	19.7°	0.39°
10	(0, 104.64, 2.14)	(0, 0.3451, 0.9385)	20.19°	19.7°	0.49°
11	(0, 164.66, 0.66)	(0, 0.2866, 0.9580)	16.88°	17°	−0.12°
12	(0, 166.18, 0.64)	(0, 0.2857, 0.9582)	16.66°	17°	−0.34°
13	(0, 167.74, 0.64)	(0, 0.2902, 0.9569)	16.63°	17°	−0.37°
14	(0, 169.41, 0.63)	(0, 0.287, 0.9576)	16.83°	17°	−0.17°
15	(0, 170.96, 0.63)	(0, 0.3451, 0.9385)	16.73°	17°	−0.27°

## Data Availability

Data available on request due to restrictions privacy.
